# A combination of spearmint and flaxseed extract improved endocrine and histomorphology of ovary in experimental PCOS

**DOI:** 10.1186/s13048-020-00633-8

**Published:** 2020-03-20

**Authors:** Mina Mehraban, Gholamali Jelodar, Farhad Rahmanifar

**Affiliations:** Department of Basic Sciences, School of veterinary medicine, Shiraz University, Shiraz, 7144169155 Iran

**Keywords:** PCOS, Sex hormones, Spearmint, Flaxseed

## Abstract

**Background:**

Polycystic ovary syndrome (PCOS) is a complex reproduction and endocrine disorder of women in the reproductive age. Spearmint (*Mentha spicata* L.) has anti-androgenic activity and flaxseed (*Linum usitatissimum* L.) contains phytoestrogen and was reported to improve PCOS conditions. This study aimed to evaluate PCOS conditions following administration of a mixture of these two plants.

**Methods:**

Twenty-four rats with regular cycles were randomly divided into four groups as control (C) and treatment-control (TC) received a combination of spearmint extract (SE) + flaxseed extract (FE). PCOS was induced in PCOS and treatment (T) groups by a single intramuscular injection of estradiol valerate. The treatment group received a combination of SE and FE for 30 days, 7 weeks after injection of estradiol valerate. Estrous cycles were monitored for 10 days and in the last day animals were sacrificed, ovaries were collected for the histomorphometric study and the serum levels of progesterone, testosterone, estradiol, and dehydroepiandrosterone (DHEA) were measured.

**Result:**

Significant rise in progesterone and a decrease in testosterone and estradiol with no significant change of DHEA in the T group, was observed in comparison with the PCOS group (*P < 0.05*). No significant difference noticed between T and control groups (C &CT) regarding evaluated hormones. A significant increase in primary, pre-antral and antral follicles noticed in the T group compared to the PCOS group. The number of cystic follicles decreased in the T group compared with the PCOS group. Granulosa layer thickness increased while the thickness of theca decreased significantly in the T group compared to the PCOS group (*P < 0.05*). No significant endocrine or histological differences noticed between C and TC groups.

**Conclusion:**

A combination of flaxseed and spearmint extract improved the endocrine profile and the histomorphometric features of the ovary in the T group compared to the PCOS group.

## Background

Polycystic ovary syndrome (PCOS) is a heterogeneous reproductive disorder in women which is associated with high levels of androgen, hyperinsulinemia and chronic anovulation, but its cause is still unknown. Endocrine, reproductive and metabolic systems are affected by this syndrome, besides it is the main cause of anovulatory infertility in women [1, 2].

Although the main etiology of this syndrome is not recognized yet, many factors such as genetics, insulin resistance, cortisol metabolism disorder, stress, and increased androgen production were reported to be involved [3, 4].

The main problem in PCOS is anovulation due to hormonal imbalance. Luteinizing hormone (LH) level is higher, but the follicle-stimulating hormone (FSH) level is lower in PCOS patients compare to normal women [5]. Follicle accumulation, absence of corpus luteum and rise in the ovarian volume are some of the remarkable signs of ovaries with PCOS [6].

Different therapeutic medicine such as metformin, clomiphene citrate, glucocorticoids and aromatase inhibitors like anastrozole have been recommended for PCOS treatment [7]. Various side effects of these medicines such as nausea, abdominal pain, and vaginal bleeding have been reported [8]. Hence, today’s uses of herbal medicines are more interested, utterly valuable, accessible and are widely used for treatment or even recuperation from diseases in some of the communities [9]. Plants contain several different families of natural products among them some have weak estrogenic or antiestrogenic activity. These compounds, termed phytoestrogens, include certain isoflavonoids, flavonoids, stilbenes, and lignans. The best-studied dietary phytoestrogens are the soy isoflavones and the flaxseed lignans [10].

Spearmint and flaxseed have been recommended and used for the treatment of PCOS. Spearmint has an anti-androgenic effect, and a significant decrease in testosterone levels was reported following the treatment of PCOS patients with spearmint [11–13].

In a case study, androgen levels in women with PCOS have decreased following consumption of spearmint hydroalcoholic extract [11]. In another study, the effects of spearmint in those who suffer from hirsutism have been revealed a decrease in FSH, LH and DHEA level [14].

Flaxseed is also recommended for the treatment of endocrine disorder and regulation of female sex hormones [15]. Administration of flaxseed hydroalcoholic extract to rat with PCOS have been reported to improve endocrine status [16]. The most important ingredients in flaxseed are fibers, minerals, vitamins, and phytoestrogen (lignan) [17, 18]. Flaxseed is an exceptionally rich source of dietary lignan. The lignan’s content of flaxseed may alter the activity of key enzymes involved in estrogen synthesis (e.g., aromatase) to modulate relative levels of circulating sex hormones and their metabolites [19, 20]. Moreover, lignan can inhibit the 5 α-reductase (the enzyme which is responsible for converting testosterone into DHT) leading to improvement of androgen levels in PCOS patients [15] Biological activities of phytoestrogens are similar to estradiol and they can bind to estradiol receptors and express estrogen effects [15, 19]. It has been proposed that consumption of flaxseed which contains a high level of lignan may facilitate the binding of testosterone in the enterohepatic circulation and increase its excretion [21] also increases levels of sex hormone-binding globulin (SHBG) thereby decreases free testosterone level [21, 22]. The phytoestrogen of flaxseed may decrease testosterone levels through negative feedback on LH [23].

Thus, by considering the anti-androgenic trait of spearmint and phytoestrogen features of flaxseed and the fact of imbalance of steroid hormones in PCOS, this study was designed to assess the role and effect of administration of a combination of these two plants on endocrine profile and histological change of ovary in estradiol valerate-induced PCOS rats.

## Methods

### Experimental design

This study was attained under the approval of the state committee on animal ethics, Shiraz University, Shiraz, Iran. Also, the testimonial of the European Council Directive (86/ 609/ EC) of November 24, 1980, regarding the standards in the protection of animals for experimental goals were followed.

Twenty-four adult female Sprague Dawley rats (200 ± 20 g) purchased from Comparative and Experimental Center of Medical Sciences Department of Shiraz Medical University. Animals with regular reproductive cycle (following 3 cycles checking), were selected and randomly dispensed into four groups as control (C) (received distill water), Treatment- Control (TC) (received 40 mg/kg hydroalcoholic extract of spearmint + 200 mg/kg flaxseed extract for 30 days by gavage). PCOS was induced in the next two groups, PCOS group, and Treatment group (T) by a single intramuscular injection of estradiol valerate (4 mg/rat). The treatment group received 40 mg/kg spearmint extract + 200 mg/kg flaxseed extract for 30 days by gavage, 7 weeks after injection of estradiol valerate, while PCOS group received distilled water during the same period. Animals were kept in the standard polypropylene cages at 20–22 °C, 38% humidity and 12/12 h light/dark cycle, fed with a standard pellet diet and had free access to tap water.

### Preparation of hydroalcoholic extract of spearmint and flaxseed

Fresh spearmint and flaxseed were purchased from a local market source in Shiraz. The plants’ qualities were confirmed by a botanist in the biology department. After clearing and drying, the spearmint was completely ground; the resulting powder was placed in 70% alcohol for 72 h. After straightening with filter paper, the rotary machine was used to concentrate extract under reduced pressure. The resultant semi-solid extract put into a lyophilizer machine for 24 h in order to make a powder. The same procedure was used for flaxseed after grinding of the seeds.

### Monitoring of reproductive cycle

**Fig. 1:**
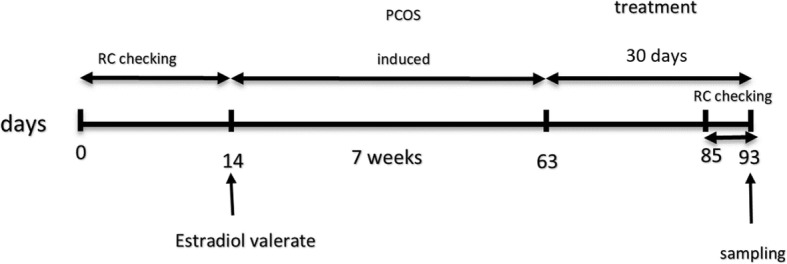
Schematic diagram of experimental design which includes, Reproductive Cycle (RC) checking, estradiol valerate injection, treatment and time of blood and tissues sampling

In rats, the reproductive cycle lasts approximately 4–5 days. The characteristic of each cycle was based on the ratio of the three types of cells which are including; cornified, leukocytes and epithelial [24]. The reproductive cycle of rats was monitored through vaginal smear according to the method described by Caligioni (2009) for two weeks before the selection of rats for the experiment (only rats with regular cycles were selected) and also was checked during the last 8 days of the experiment (from day 85 of the experiment) (Fig. 1).

### Induction of PCOS and treatment

PCOS was induced in two groups (treatment and PCOS groups) of rats with regular estrous cycles 7 weeks after a single intramuscular injection of 4 mg of estradiol valerate/rat dissolved in 0.2 ml of sesame oil [25, 26].

### Blood sampling and histological study

During the last week of treatment, again the reproductive cycle of all rats assessed. On day 93 (the last day of the experiment) rats were anesthetized by 2% diethyl ether-saturated cotton ball in a chamber for 3–5 min., and euthanized by whole blood collection through heart puncture, and sera were used for measuring hormones including estrogen, progesterone, testosterone, and dehydroepiandrosterone (DHEA).

Ovaries were dissected out and subjected to histomorphometric study after fixation and tissue processing. Serial sections of ovaries were prepared at a thickness of 7 μm and stained with hematoxylin and eosin and the same section of all ovaries was selected for histological study. Different types of follicles including; primary, preantral, antral and cystic were evaluated under a light microscope (CX21 OLYMPUS, Japan) and were photographed by an adjusted digital camera (AM423U Eyepiece camera, Dino- Eye Taiwan). The follicles were characterized as primary (oocytes with one layer of cuboidal granulosa cells), preantral (oocytes with 2–5 layer of cuboidal granulosa cells), antral (oocyte surrounded with more than 5 layer of granulosa cells and small or large area of follicular fluid) and atretic cyst-like follicles (large fluid-filled cyst with an attenuated granulosa cell layer, and a disconnected oocyte from granulosa cells. Granulosa and theca layer in different follicles were measured also follicle diameter was evaluated. Furthermore, the number of primary, preantral, antral and cystic follicles was measured by Dino Capture 2.0.

### Hormone assay

The hormones including testosterone, estrogen, progesterone, and DHEA in serum were measured by the ELISA method using ELISA reader (Monobind, Inc. lake Forest, CA (92630) USA.

### Statistical analysis

All results are presented as Mean ± standard error of the mean (Mean ± SEM). Statistical analysis was performed by (SPSS-24.0). Statistical differences between groups were compared with (one-way ANOVA). Pair group comparison was measured by the LSD test. Statistical significance was set at *P < 0.05*.

## Results

### Reproductive cycle

Following induction of PCOS mean body weight of this group increased significantly compare to contril groups, andt treatment with the extract improved their weight (Table 1). The results of reproductive monitoring showed that after induction of PCOS with estradiol valerate the reproductive cycle of animals was stopped in the estrus stage, and following treatment with extract it has been started but with a longer period (Table 2).

**Table 1:** Changes in body weight (g) mean ± SEM during the experimental period

	control	Treatment control	PCOS	Treatment
Initial weight (g)	192.10 ± 4.32 a	189.71 ± 4.51 a	194.20 ± 3.41 a	190.11 ± 4.75 a
After induction of PCOS	205.31 ± 5.92 a	197.41 ± 3.98 a	232.60 ± 8.45 b	227.30 ± 8.69 b
Last day of experiment	214.9 ± 6.12 a	210.41 ± 7.61 a	238.50 ± 9.40 b	220.40 ± 8.05 ba

Different alphabet indicate a statistically significant difference between groups (*P* < 0.05).

**Table 2:** Duration of mean ± SEM of estrous cycles (day) in four different groups during the experimental period

	control	Treatment control	PCOS	Treatment
Beginning	4.9 ± 0.19 a	4.4 ± 0.20 a	4.7 ± 0.35 a	4.6 ± 0.27 a
After Injection of estradiol valerate	4.6 ± 0.14 a	4.2 ± 0.23 a	-- b	-- b
After treatment	4.4 ± 0.18 a	4.6 ± 0.25 a	-- b	4.8 ± 0.34 a

Different alphabet indicate a statistically significant difference between groups (*P* < 0.05).

### Changes of sex hormones in the serum of different groups

**Fig. 2:**
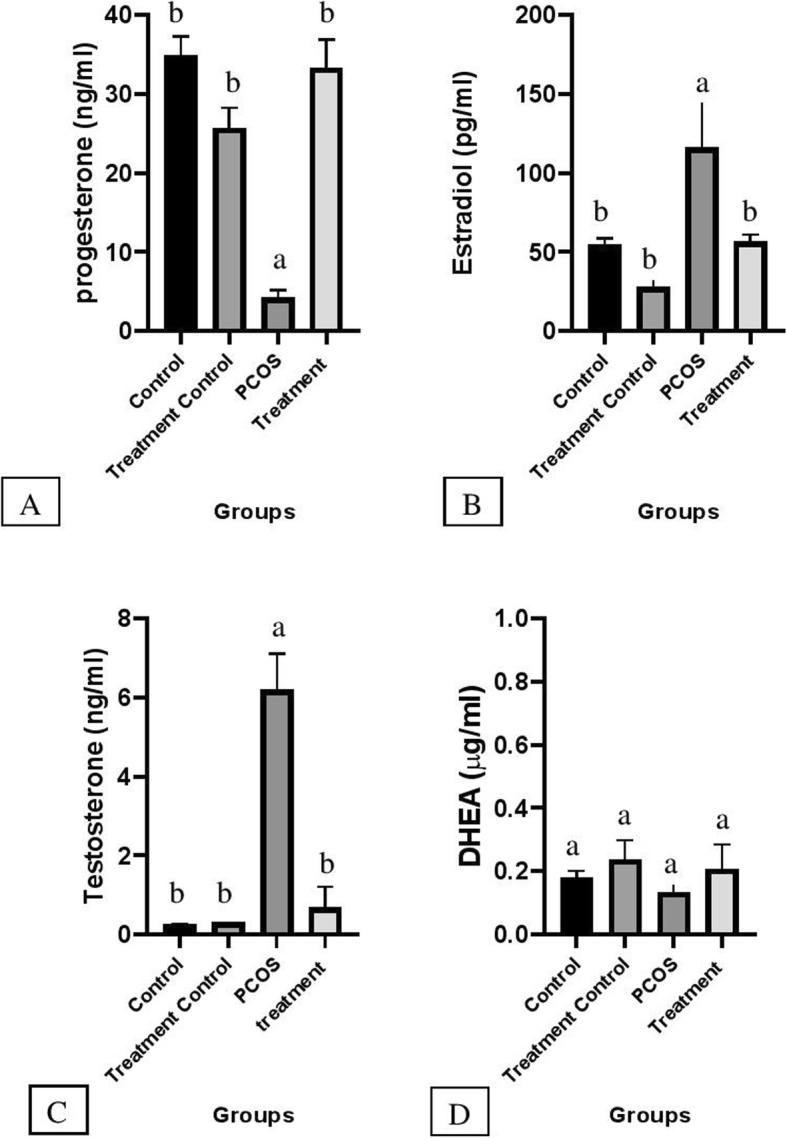
Illustrated mean ± SEM level of Progesterone (**a**), Estradiol (**b**), Testosterone (**c**) and DHEA (**d**) in serum and effects of flaxseed and spearmint combined treatement on hormones given in four different groups. Different alphabet indicate a statistical significant difference between groups (*P* < 0.05)

The mean concentration of progesterone hormone (Fig. 2) in the treatment group increased significantly in comparison with PCOS (*P < 0.05)* but, there was no significant difference between treatment, treatment control, and control groups. The level of estradiol hormone in the PCOS group elevated compared with other groups (*P < 0.05*) but, the mean concentration of this hormone in the treatment group declined compared with the PCOS group significantly (*P < 0.05)*. There was no marked difference in estradiol level between control, treatment control, and treatment groups.

Mean serum testosterone concentration in the PCOS group increased significantly in comparison with other groups (*P < 0.05*). However, the mean concentration of this hormone has not changed remarkably in C, TC, and T groups and there was no significant difference between these groups.

As is illustrated in Fig. 2 C &B level of estradiol and testosterone in the PCOS group is dramatically higher than that of other groups. However, in the treatment group, testosterone and estradiol levels decreased significantly (*P < 0.05*). Moreover, the progesterone level in the treatment group has increased compared with the PCOS group but the DHEA level did not change significantly among the groups (Fig. 2 A & D).

### Ovarian histomorphology

The number of cystic follicles in the PCOS group increased significantly compared to other groups (Table 3). The average number of the primary and preantral follicles in the PCOS group decreased compared to others and it was statistically significant with TC and C groups. The numbers of antral follicles in the PCOS group decreased significantly in comparison with other groups.

**Table 3:** Comparison of mean ± SEM follicle’s numbers between groups (*n* = 6)

Groups	Primary F	Pre- antral F	Antral F	Cystic F
Control	35.01 ± 1.63 ^a^	41.2 ± 1.38^a^	11.92 ± 0.335^a^	0^c^
Treatment control	38.16 ± 2.19^a^	42.27 ± 1.89^a^	8.08 ± 0.48^a^	0^c^
PCOS	14.16 ± 0.87^b^	26.05 ± 0.96^b^	1.83 ± 0.30^b^	4.66 ± 1.02^a^
Treatment	18.83 ± 1.66^b^	29.13 ± 1.38^b^	4.25 ± 0.32^c^	1.5 ± 0.56^b^

Different alphabet indicate the statistically significant difference between groups (*P* < 0.05).

**Fig. 3:**
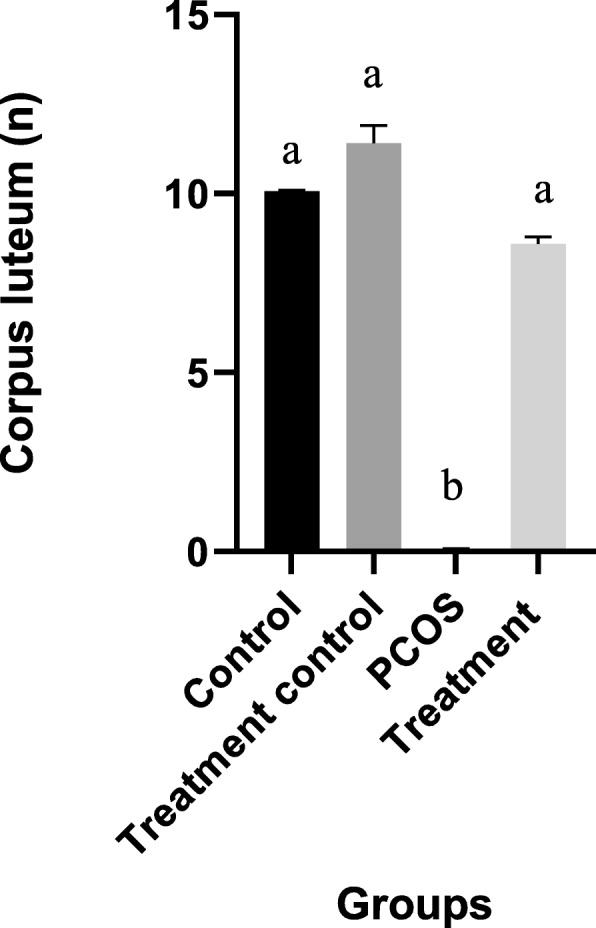
Illustrated mean ± SEM of Corpus luteum in four different groups. Different alphabet indicate a statistically significant difference between groups (*P* < 0.05)

The cystic follicle in the PCOS condition is characterized by an increase of thickness of tunica albuginea as a result of an increased level of collagen and hypertrophy and hyperplasia of theca layer and stromal fibrosis. The number of cystic follicles in the T group decreased significantly in comparison with the PCOS group. No cystic follicle was observed in treatment control and control groups. Mean number of corpus luteum is presented in the Fig. 3. There was no corpus luteum on the ovary of the PCOS group.

**Fig. 4:**
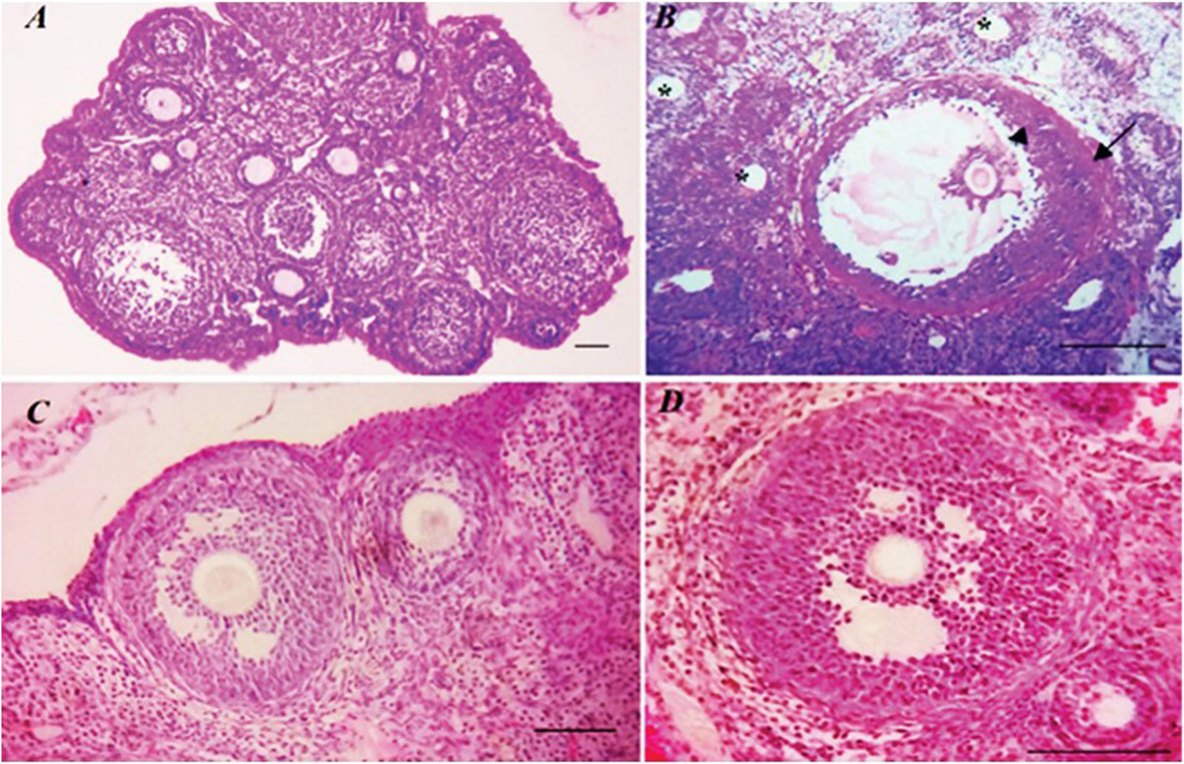
Depicts ovaries section in 4 groups, PCOS (**a**) with cystic follicles 10.0 μm, Treatment (**b**) with pre-antral and antral follicles, Control (**c**) including antral follicles and Treatment Control (**d**) which includes primary follicles as well as antral follicles (H&E staining) index bars, 50.0 μm

**Fig. 5:**
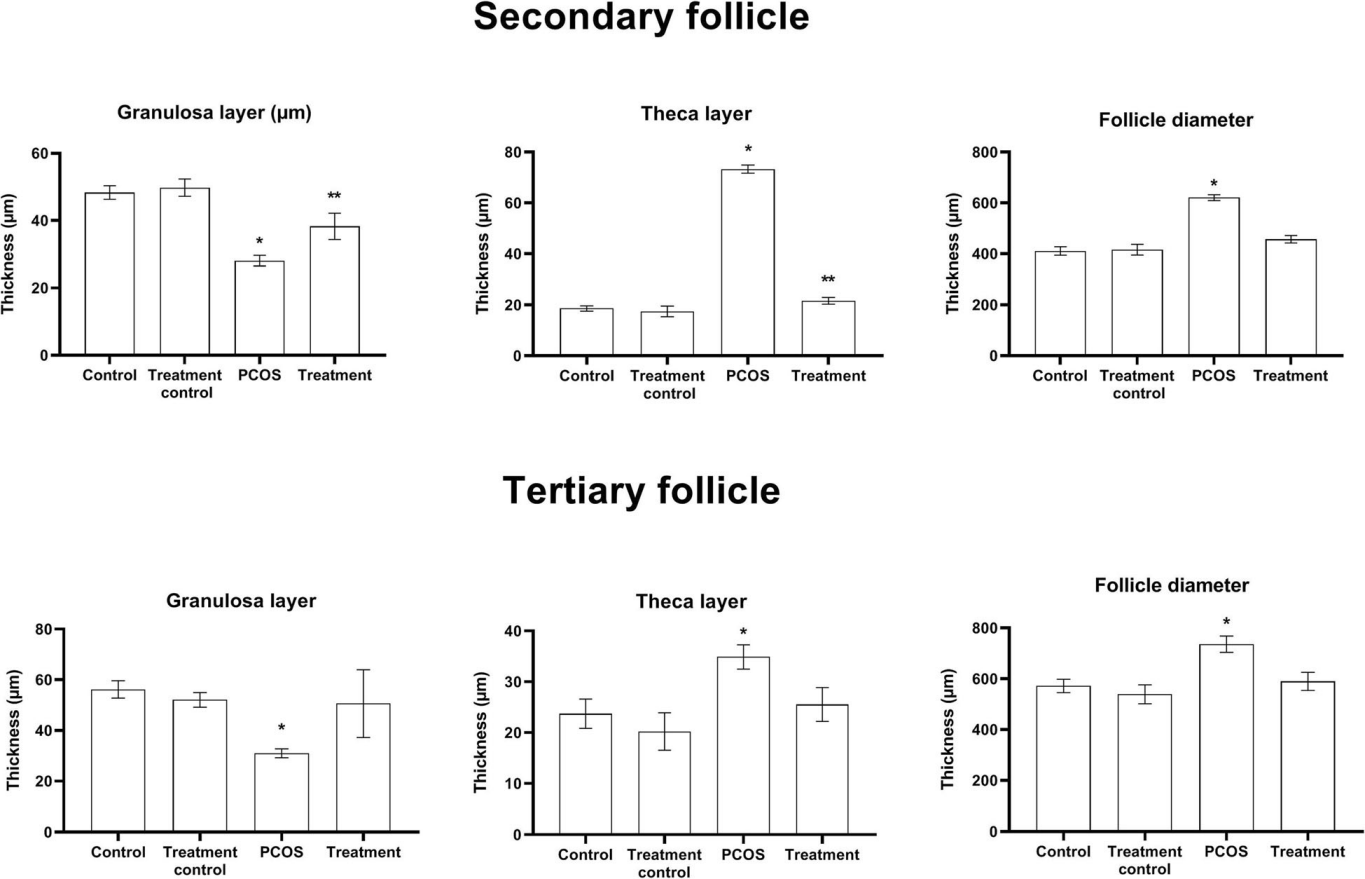
Value presented as mean ± SEM of follicular parameters in the secondary and tertiary follicles (*n* = 6). * and ** significant different with other groups (0.05)

Histological micrograph from ovaries of different groups is presented in Fig. 4. The thickness of the granulosa layer in secondary follicles decreased significantly while the cause is of the theca layer of these follicles increased in the PCOS group compared to other groups *(P < 0.05*). This value also showed a significant increase in the treatment group compared with the treatment control and control groups *(P < 0.05*). Significantly increases of secondary follicle diameter were observed in the PCOS group compared with other groups (Fig. 5) (*P < 0.05*).

## Discussion

Spearmint and flaxseed have individually been recommended for treatment or improving PCOS in women [11, 14, 15]. In this study, we investigate the importance of using a combination of flaxseed and spearmint hydroalcoholic extract on PCOS improvement in a rat model.

We exploited estradiol valerate to induce PCOS. As is illustrated, testosterone and estradiol levels in the PCOS group increase dramatically compared to the control group while progesterone levels in the PCOS group decreased significantly. However, there wasn’t a significant difference in the DHEA level between groups. Similar results were reported by other researchers following induction of PCOS using estradiol valerate [16, 27]. Progesterone levels in the treatment group increased significantly in comparison with the PCOS group and this increase was slightly higher than using flaxseed or spearmint treatment lonely [16]. Administration of hydroalcoholic flaxseed extract to immature rats has been reported to increase progesterone levels significantly [28]. It seems that using a combination of these two extracts has the potential to improve the ovarian function, leading to ovulation and develop of corpus luteum as the source of progesterone.

In the current study level of testosterone decreased tremendously in the PCOS rats treated with a combination of spearmint and flaxseed extracts, which is four times lower than our previous report when flaxseed was administrated alone [16]. The reason behind it may be due to the anti-androgenic effect of spearmint and phytoestrogen action of flaxseed [11, 17] which can lower the amount of free testosterone [11, 14, 15]. Similarly, a significant decrease in testosterone level was reported following a 12-weeks treatment of forty-eight postmenopausal women with flaxseed [29]. Moreover, the administration of spearmint as an anti-androgenic source to PCOS-induced rats for 20 days was reported to decrease the level of testosterone significantly [30].

The level of estradiol also decreased significantly in the treatment group received a combination of flaxseed and spearmint extract in comparison with the PCOS group. This decrease exceeded from the previous report using flaxseed alone [16].

The decrease in the level of estradiol could be due to the presence of lignan in flaxseed that may lead to a rise in SHBG production which in turn decreases the level of free estradiol [19]. Moreover, lignan can inhibit aromatase activity thereby decrease estradiol production. Other researches have reported the same results in menopause women [31].

There was no significant change in DHEA between groups. The same results were observed in other studies [14, 16, 31]. Since the main source of DHEA is the adrenal gland [32] its secretion has not been affected during the induction of PCOS or treatment with these extracts.

Following induction of PCOS number of primary, preantral and antral follicles decreased, while the number of cystic follicles increased significantly. Treatment of rats with a combination of flaxseed and spearmint extract improved these undesired alterations. Similar histological changes were reported following the induction of PCOS in female rats [33] and treatment with flaxseed extract [16].

Granulosa thickness increased while thicknesses of theca layer decreased in the T group compared to the PCOS group. Granulosa proliferation is regulated by many paracrine/endocrine factors such as follicular estrogen concentration, growth factors, and TGF-β. GDF9 is a member of the TGF-β family that originates from the oocyte and is essential for normal growth and development of ovarian follicles. The dominance of follicular concentration of estrogen and FSH are essential for continued granulosa cell growth and accumulation [34]. Hence the combination of these extracts has improved the follicular situation possibly through any of these mechanisms (endocrine /paracrine), which need further investigation.

## Conclusion

In summery administration of a combination of spearmint and flaxseed extract to PCOS animals improved endocrine secreation including estradiol, progesterone and testosterone level and ovarian histology which are more remarkable than using flaxseed or spearmint alone, reported previously. The results highlighted the potential effects of using a combination of spearmint and flaxseed extract for treatment of PCOS.

## Supplementary information

**Additional file 1.** Presentation of follicles number in the ovary as percentage.

## Data Availability

Data will be made available from the corresponding author on request.
