# Supplementary material for: A combination of spearmint and flaxseed extract improved endocrine and histomorphology of ovary in experimental PCOS
Source: J Ovarian Res. 2020 Mar 20;13:32. doi: 10.1186/s13048-020-00633-8 (PMC7085145; doi:10.1186/s13048-020-00633-8)
Supplement: Supplementary file 1 — Additional file 1. Presentation of follicles number in the ovary as percentage. [file 13048_2020_633_MOESM1_ESM.docx]

Additional files

File 1: Presentation of follicles number in the ovary as percentage.

|  | **Primary F** | **Pre-antral F** | **Antral F** | **Cystic F** |
| --- | --- | --- | --- | --- |
| **Control** | 39.72 ^a^ | 46.74 %^a^ | 13.50 %^a^ | 0^a^ |
| **Control Treatment** | 43.11 %^a^ | 47.75 %^a^ | 9.12 %^a^ | 0^a^ |
| **PCOS** | 30.45 %^b^ | 56.02 %^b^ | 3.93 %^b^ | 4.66 %^b^ |
| **Treatment** | 35.05 %^b^ | 54.23 %^b^ | 7.91 %^c^ | 2.79 %^c^ |

Table3 Comparison of the percentage of different type of follicles between groups (n=6)

Different alphabet indicate the statistically significant difference between groups (P<0.05)
